# A catalog of GWAS fine-mapping efforts in autoimmune disease

**DOI:** 10.1016/j.ajhg.2021.03.009

**Published:** 2021-04-01

**Authors:** Minal Caliskan, Christopher D. Brown, Joseph C. Maranville

**Affiliations:** 1 Department of Informatics and Predictive Sciences, Bristol Myers Squibb, Princeton, NJ 08540, USA; 2 Department of Genetics, Perelman School of Medicine, University of Pennsylvania, Philadelphia, PA 19104, USA

## Abstract

Genome-wide association studies (GWASs) have enabled unbiased identification of genetic loci contributing to common complex diseases. Because GWAS loci often harbor many variants and genes, it remains a major challenge to move from GWASs’ statistical associations to the identification of causal variants and genes that underlie these association signals. Researchers have applied many statistical and functional fine-mapping strategies to prioritize genetic variants and genes as potential candidates. There is no gold standard in fine-mapping approaches, but consistent results across different approaches can improve confidence in the fine-mapping findings. Here, we combined text mining with a systematic review and formed a catalog of 85 studies with evidence of fine mapping for at least one autoimmune GWAS locus. Across all fine-mapping studies, we compiled 230 GWAS loci with allelic heterogeneity estimates and predictions of causal variants and trait-relevant genes. These 230 loci included 455 combinations of locus-by-disease association signals with 15 autoimmune diseases. Using these estimates, we assessed the probability of mediating disease risk associations across genes in GWAS loci and identified robust signals of causal disease biology. We predict that this comprehensive catalog of GWAS fine-mapping efforts in autoimmune disease will greatly help distill the plethora of information in the field and inform therapeutic strategies.

## Introduction

**Figure 1:**
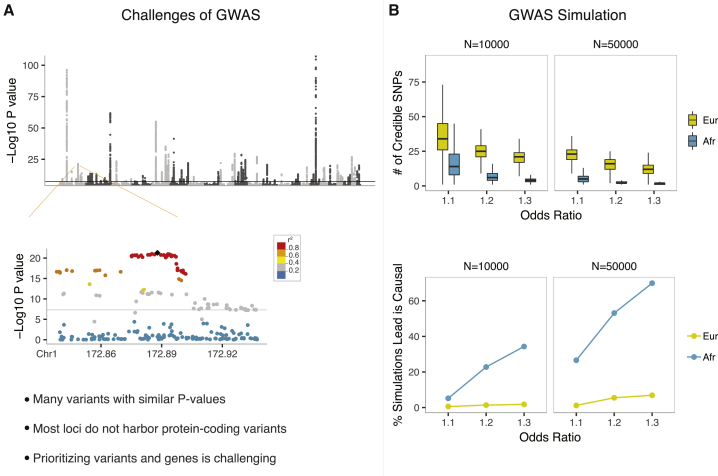
Challenges of GWASs (A) An example Manhattan plot displaying Crohn disease GWAS p values.^3^ The zoomed-in plot of the chr 1q24.3 locus^3^ illustrates the correlation between variants (r^2^) and association p values. (B) The upper figure shows the number of statistically prioritized (95% credible SNP set) variants based on 1,000 simulations under different odds-ratio, sample-size, and ethnicity scenarios. The lower figure shows the percentage of simulations that identified the assigned causal variant as the lead variant. Haplotypes were constructed from 1000 Genomes European (CEU, TSI, FIN, GBR, and IBS) or African (YRI, LWK, GWD, MSL, ESN, ASW, and ACB) subjects. Common genetic variants (MAF > 0.01) across 100 kb region in the chr 1q24.3 locus shown in Figure 1A were included in haplotype construction. Among observed credible SNP set variants, a randomly chosen SNP was assigned as the causal variant for simulation purposes. GWAS p values were simulated with the simGWAS package^4^ under different odds-ratio, sample-size, and ethnicity scenarios. 95% credible SNP sets were calculated as implemented in the coloc package.^5^ Numbers of credible SNP set variants in each simulation and the percentage of simulations that identified the assigned causal SNP as the lead SNP were plotted.

Over the past 15 years, genome-wide association studies (GWASs) have identified thousands of loci that are associated with complex traits and common diseases.^1^ Because genotypes at nearby variants are usually correlated, a phenomenon referred to as linkage disequilibrium (LD), significant GWAS findings pinpoint genomic regions rather than individual variants (Figure 1A). The resolution at individual loci is influenced by many factors, including the effect size and minor-allele frequency (MAF) of causal variants and local recombination rates, as well as the sample size and ethnic composition of study cohorts (Figure 1B). Additionally, nearly 90% of significant GWAS variants reside in non-protein-coding regions of the genome,^1^^,^^2^ making their functional interpretation challenging.

While GWASs continue to identify genomic loci associated with complex traits, tremendous efforts go into dissecting such loci to identify trait-relevant genes, regulatory regions, and variants.^6^, ^7^, ^8^, ^9^ This process of identifying trait-relevant genetic elements in an already constrained genetic locus is referred to as “fine mapping” and is crucial to understanding the underlying disease mechanisms and translating GWAS findings into potential therapeutics. Drug targets that are genetically supported have a significantly higher probability of being approved as successful treatments.^10^^,^^11^ Many established autoimmune-disease drug targets, such as TNF blockers, IL23/IL12 antagonists, and CTLA4 agonists, have either prospectively or retrospectively been shown to reside in GWAS loci of autoimmune diseases.^1^ With hundreds of loci having been associated with autoimmune diseases, comparative analyses of GWAS follow-up studies have great potential to reveal the most consistently dissected loci where a single gene was prioritized on the basis of multiple sources of functional and statistical evidence. Such approaches could then be integrated into the drug-discovery pipelines to allocate time and resources into the most probable targets.

Fine mapping is also critical to understanding the pleiotropic effects of genes across a range of phenotypes. For example, there is notable overlap in GWAS loci across diseases that involve autoimmunity.^12^^,^^13^ However, the presence of a shared GWAS locus does not imply the same underlying causal variant or the same gene. There are also several reports of shared GWAS loci that harbor alleles with opposite effects on different autoimmune diseases.^14^^,^^15^ Comparative analyses of GWAS fine-mapping studies could highlight both shared and disease-specific causal variants, genes, and pathways that are involved in autoimmunity. Insight gained from these analyses could aid development of therapeutic hypotheses and experimental directions that could be pursued within or across autoimmune diseases.

Characterized by an inappropriate and excessive inflammatory response against self-antigens, common autoimmune diseases include Crohn disease (CD; MIM: 266600), ulcerative colitis (UC; MIM: 191390), rheumatoid arthritis (RA; MIM: 180300), juvenile idiopathic arthritis (JIA; MIM: 618795), psoriasis (PSO; MIM: 177900), type 1 diabetes (T1D; MIM: 222100), multiple sclerosis (MS; MIM: 126200), vitiligo (VIT; MIM: 193200), systemic lupus erythematosus (SLE; MIM: 152700), and systemic sclerosis (SS; MIM: 181750). Dozens to hundreds of GWAS loci are associated with each of these autoimmune diseases, yet there has not been a comprehensive study summarizing results from GWAS fine-mapping efforts in autoimmunity. Here, we used a systematic approach and formed a catalog of published studies with evidence of fine mapping for at least one autoimmune GWAS locus. We summarized the lessons learned about the genetic architecture of complex autoimmune diseases, highlighted some of the inconsistencies that are yet to be resolved in the literature, and used a novel approach to prioritize candidate trait-relevant genes in GWAS loci on the basis of both genome-wide and locus-specific fine-mapping evidence.

## A catalog of autoimmune GWAS fine-mapping efforts

**Figure 2:**
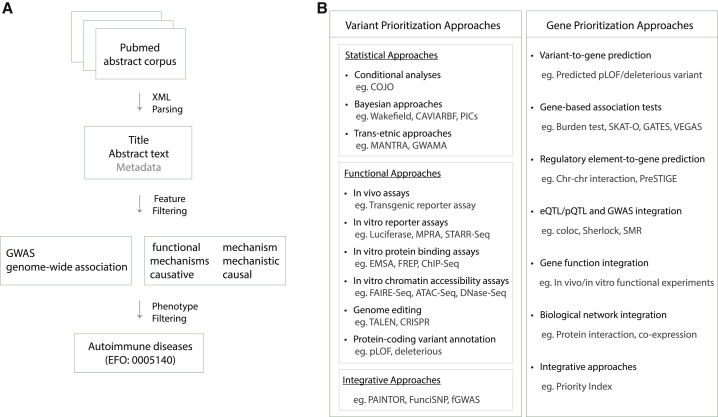
A catalog of autoimmune GWAS fine-mapping efforts (A) Approach used to identify manuscripts that are most likely on the autoimmune GWAS fine-mapping topic. PubMed abstracts published between January 2005 and January 2020 were retrieved from the National Library of Medicine. There were 9,744 manuscripts following feature (GWAS and fine-mapping terms) filtering and 837 manuscripts following autoimmune disease filtering. (B) The list of causal-variant and gene-prioritization approaches that were assessed during review and identification of autoimmune GWAS fine-mapping studies.

We put together a list of 22 manuscripts that are commonly cited as examples of “GWAS fine-mapping studies” (Table S1). Using these 22 manuscripts, we defined a set of informative terms that often co-occur in the abstracts of the manuscripts on GWAS fine-mapping efforts. We found that in 18 of these 22 abstracts either the “GWAS” or “genome-wide association” term co-occurs with one of the following six terms: “functional,” “causal,” “mechanisms,” “causative,” “mechanism,” or “mechanistic.” Accordingly, we downloaded PubMed abstracts from the National Library of Medicine and searched all abstracts published between January 2005 and January 2020 for the co-occurrence of “GWAS”/“genome-wide association” with any of the aforementioned six terms (Figure 2A). We further filtered the abstracts to retrieve those that contained one or more autoimmune disease ID (diseases under “experimental factor ontology” (EFO) autoimmune disease category: 0005140) shown in Table S2. After performing these two searches sequentially, we identified a total of 837 manuscripts that most likely describe autoimmune GWAS fine-mapping studies. We manually reviewed these 837 manuscripts and assessed whether they include results based on at least one of the variant- or gene-prioritization approaches shown in Figure 2B. For statistical variant-prioritization approaches, we additionally required that the manuscripts fulfill at least one of the following two criteria: (1) deep resequencing followed by genotyping or imputation of discovered variants in the same ancestry population and (2) dense genotyping in a population that matches the ancestry of the population for which the genotyping array was optimized. The list of fine-mapping approaches we used (Figure 2B) have been previously described and discussed in detail by a number of review papers on GWAS fine-mapping approaches.^6^, ^7^, ^8^, ^9^ In total, we cataloged 85 manuscripts with evidence of fine mapping for at least one autoimmune GWAS locus (Table S3). There were seven (CD, UC, IBD, RA, PSO, T1D, and MS), 11 (CD, UC, IBD, RA, JIA, PSO, T1D, VIT, MS, SLE, and SS), and 15 (CD, UC, IBD, RA, JIA, PSO, T1D, MS, SLE, SS, VIT, Behcet disease [BD; MIM: 109650], psoriatic arthritis [PSA; MIM: 607507], Graves disease [GD; MIM: 275000], and ankylosing spondylitis [AS; MIM: 106300]) autoimmune diseases with allelic heterogeneity, variant prioritization, and gene-prioritization estimates, respectively (Tables S5, S6, and S8).

## 22% of complex autoimmune-disease-associated loci display allelic heterogeneity

A critical step in fine mapping is to address allelic heterogeneity, i.e., the presence of multiple independent variants that affect the function of the same gene and lead to the same phenotype. Allelic heterogeneity is common for Mendelian diseases, which are caused by rare (risk-allele frequency < 1% in the general population), highly penetrant variants. For example, the ClinVar database^16^ includes 34 pathogenic *AIRE* (MIM: 607358) variants that cause polyglandular autoimmune syndrome and 16 pathogenic *FAS* (MIM: 134637) variants that cause autoimmune lymphoproliferative syndrome (Table S4). In fact, allelic heterogeneity was reported for 74% of the Mendelian-disease-causing genes in the ClinVar database (Table S4).

Linkage disequilibrium complicates efforts to assess allelic heterogeneity for common variants associated with complex diseases. Researchers have developed three categories of methods to address this challenge: conditional analyses, haplotype-association tests, and Bayesian approaches. In conditional analyses, independence of a variant is tested after it is conditioned on the lead variant in the locus; if a nearby variant remains significant, it is considered as having a distinct additional signal. Haplotype-based association tests directly model combinations of alleles at multiple variants. In so doing, they can help researchers to understand the effect sizes and directions of the variants that are not fully independent from each other. Bayesian approaches use model selection strategies to identify independent signals of association within the same locus, leveraging the distribution of effect sizes and local patterns of linkage disequilibrium from reference panels. Here, through our systematic review we compiled allelic heterogeneity estimates for 129 loci associated with autoimmune disease.^17^, ^18^, ^19^, ^20^, ^21^, ^22^, ^23^, ^24^, ^25^ Across the 129 loci examined, 28 (22%) were reported to have more than one signal based on at least one study (Table S5).

**Figure 3:**
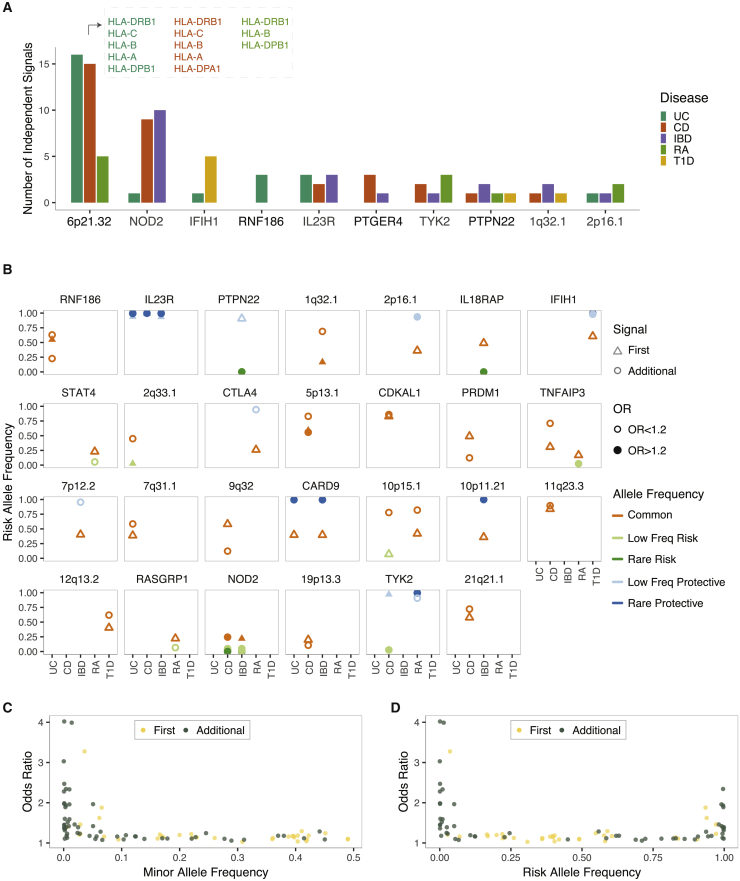
The extent of allelic heterogeneity in complex autoimmune diseases (A) Example GWAS loci where allelic heterogeneity was reported for at least one autoimmune disease. (B) Risk-allele frequency and effect size of the first and additional signals in non-HLA loci with allelic heterogeneity. Gene names are included only when a single gene was predominantly reported as the candidate trait-relevant gene in the literature. Note that the HLA locus is not included because of the complexity of the locus and that there are inconsistencies in how genetic associations in this locus were reported (i.e, reporting was at the haplotype level, amino acid level, and nucleotide level). Common-allele frequency (AF), AF > 0.1; low-allele frequency, 0.01 < AF < 0.1; and rare-allele frequency, AF < 0.01. Abbreviations are as follows: UC, ulcerative colitis; CD, Crohn disease; IBD, inflammatory bowel disease; RA, rheumatoid arthritis; T1D, and type 1 diabetes. (C) The relationship between odds ratios and minor-allele frequencies. (D) The relationship between odds ratios and risk-allele frequencies.

Expectedly, the HLA locus (chr 6p21.32), known for its extreme polymorphism, extensive LD, and high gene content, was reported to display the largest number of independent signals associated with complex autoimmune diseases (Figure 3A). This locus was exceptional in that independent signals identified were common, large-effect-size protein-coding variants that act through multiple different genes in the locus. Although evidence for multiple independent autoimmune-disease signals in the HLA locus is mounting, results from this locus are particularly difficult to summarize because there is no consensus on how genetic variations in this locus should be captured and reported. The four-digit “classical HLA alleles,” for instance, consider each unique protein sequence as a different “allele” and provide haplotype-level associations. Amino-acid-based models test each variable amino acid individually and provide a finer-scale resolution of the association signals. However, the complex nature of the HLA locus challenges the interpretation of the amino-acid-based models as well because a large fraction of amino acid positions in this locus are multi-allelic, and association p values are influenced by both the degree of phenotypic association and the level of complexity at each position.^21^ For these reasons, despite the importance of the HLA locus in autoimmune diseases, throughout the rest of the manuscript we will focus primarily on results from the non-HLA loci. Among non-HLA loci with allelic heterogeneity, the NOD2 locus (chr 16q12.1) contained the largest number of independent signals associated with a complex autoimmune disease (Figures 3A and 3B). This locus as well had unique properties in that it also contained low-frequency, large-effect-size protein-coding association signals that had high penetrance.^26^ In addition to the protein-coding variants with predicted deleterious effects (rs2066845, rs2066844, rs104895444, and rs104895467), the NOD2 locus also includes independent common non-coding variants (rs2357623 and rs72796367) and other rare variants with predicted benign (rs5743271) or unknown (rs184788345) effects that are associated with Crohn disease. In fact in most loci with allelic heterogeneity, there was at least one signal that was driven by a non-coding variant (Table S5), necessitating further gene-prioritization approaches to dissect whether all independent signals impact a single gene or whether multiple variants that act through different genes in the same locus contribute to the same disease. Given that the immune genes tend to cluster together in the human genome,^27^ the latter possibility should also be considered while following up on these signals experimentally. Mendelian randomization approaches or eQTL-GWAS colocalization analyses that allow more than one causal variant in any given locus could also help with addressing this question.

We also noted that additional independent signals identified in conditional analyses were enriched to represent rare- or low-frequency variants with larger effect sizes (mean MAF = 0.09, mean risk-allele odds ratio = 1.52) than those of the lead variants (mean MAF = 0.25, mean risk-allele odds ratio = 1.26). the Welch’s t test p value was 3.8 × 10^−6^ for MAF comparison and 0.018 for the odds-ratio comparison (Figure 3C and Table S5). Although in fewer loci, common variants have also been identified as additional signals in autoimmune-disease loci (Figures 3B–3D). Allelic heterogeneity is expected to hamper complex-disease-association signals as multiple trait-relevant variants with distinct phenotypic effects may cancel out each other’s signals when population-based association studies are performed.^28^^,^^29^ In essence, for loci that could be identified through GWAS, one population genetics theory predicts that there is likely negligible allelic heterogeneity or that the risk allele frequency/effect size of the predominant signal is significantly different than those of additional signals in the locus.^28^^,^^29^ Alternatively, the depletion of allelic heterogeneity driven by multiple independent common variants could be due to the presence of several causal variants that are in high LD. It is important to note that when several causal variants are correlated, statistical approaches fail to differentiate between their effects, but we cannot rule out the existence of allelic heterogeneity either.

## Causal variants and the extent of genetic overlap between different autoimmune diseases

Even when all variants in a locus are included in an association study, there is a reasonable chance that, as a result of statistical fluctuations, the lead variant representing each independent signal is not the true causal variant. For example, we simulated GWAS p values in a randomly chosen Crohn disease GWAS locus under different odds-ratio, sample-size, and ethnicity scenarios. Even in the “best-case” scenario where the association signal was driven by a relatively large-effect variant (OR = 1.3) in a well-powered study (n = 50,000, 1:1 case-control ratio) from an African American population sample (with expected small LD windows), there was only a 70% chance that the lead variant identified was the true causal variant (Figure 1B).

Given the observed pattern of association in a locus, Bayesian approaches can help to infer the probability that a variant is causal and can output a list of variants that include the causal variant with a certain probability threshold. Additionally, functional experiments including *in vivo* transgenic reporter assays; *in vitro* reporter, protein binding, and chromatin-accessibility assays; genome editing tools; and algorithms that integrate both functional annotations and statistical approaches could help prioritize the causal variants in loci associated with complex diseases. There is no gold standard in variant fine-mapping strategies, but consistent results across different studies and approaches can improve confidence in the fine-mapping findings.

**Figure 4:**
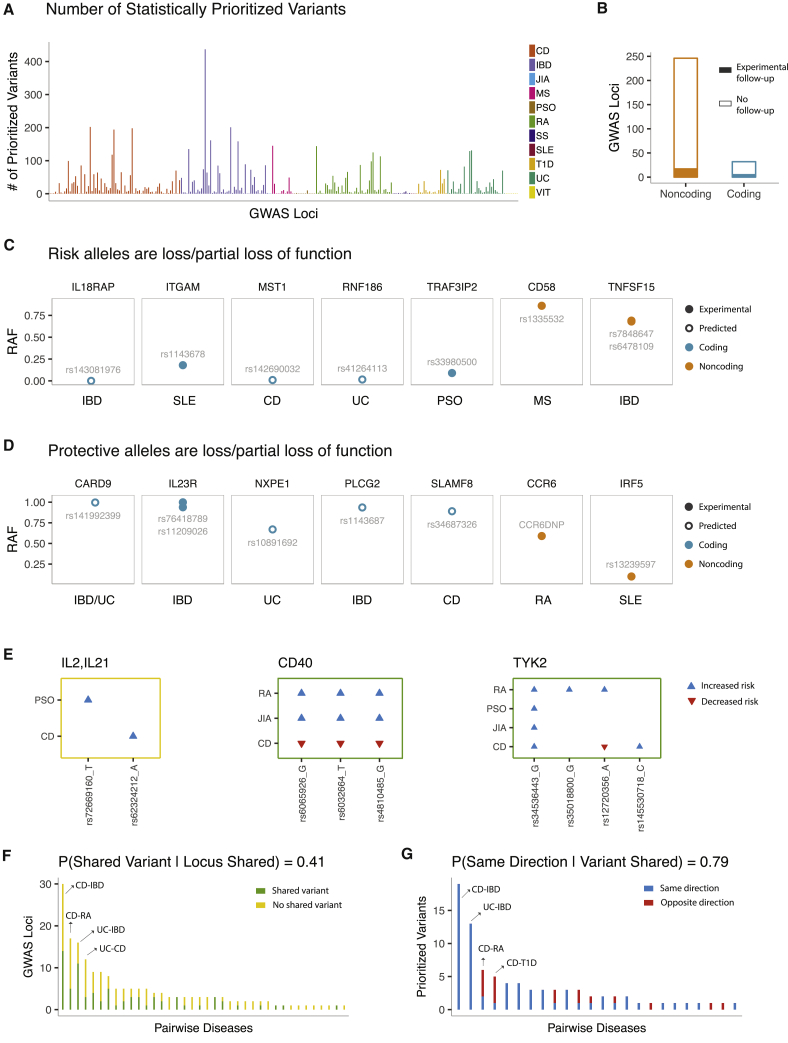
An overview of the candidate causal variants in autoimmune GWAS loci (A) Number of prioritized variants based on statistical estimates across all locus-by-disease combinations. (B) Locus-by-disease combinations stratified by whether a predicted damaging protein-coding variant was prioritized (n = 32) or not (n = 246). 22 signals (7 protein-coding, 15 non-coding) in total were experimentally followed up on. (C) Example gene-disease pairs where loss- or partial-loss-of-function alleles increase risk of autoimmune disease. (D) Example gene-disease pairs where loss- or partial-loss-of-function alleles protect against autoimmune disease. (E) Example loci with discordance in the set of prioritized variant(s) or direction of genotype effect on the risk of different autoimmune diseases. (F) The x axis shows the 38 pairwise autoimmune disease combinations for which at least one GWAS locus is shared and fine-mapped for both diseases. The y axis shows the number of loci that harbor at least one shared prioritized variant (green) versus the number of loci that do not harbor any shared prioritized variant (yellow). (G) The x axis shows the 24 pairwise autoimmune disease combinations that share at least one prioritized variant in at least one locus. The y axis shows the number of shared variant(s) that have the same direction of effect on both diseases (blue) versus the number of variants with opposite direction of effect on each disease (red). Note that when multiple prioritized variants overlapped for two diseases, only one variant per each independent association signal was included during assessment of the direction of effect.

Here we compiled 164 autoimmune-disease loci (Table S6) for which the causal variants were prioritized via at least one of the fine-mapping approaches shown in Figure 2. These 164 loci included 278 combinations of locus-by-disease association signals with 11 autoimmune diseases. The vast majority of the association signals (n = 257, 92.4%) were fine-mapped via statistical approaches.^3^^,^^2^^,^^17^, ^18^, ^19^, ^20^^,^^22^, ^23^, ^24^, ^25^^,^^30^^,^^31^ A median of eight variants were prioritized on the basis of statistical estimates across all locus-by-disease combinations (Figure 4A). Only 22 (7.9%) signals were fine-mapped via functional experiments,^32^, ^33^, ^34^, ^35^, ^36^, ^37^, ^38^, ^39^, ^40^, ^41^, ^42^, ^43^, ^44^, ^45^, ^46^, ^47^, ^48^ and 17 (6.1%) were fine-mapped via integrative approaches.^49^^,^^50^

At any disease-associated variant, the disease risk allele might increase or decrease the function of the target gene or might lead to acquisition of a new function. Elucidating the mechanism of action of the risk and protective alleles could help researchers to understand how target genes are affected and how they should be modulated in patient cohorts to reduce or eliminate the disease symptoms. Among fine-mapped autoimmune-disease signals, 32 (11.5%) included at least one predicted pLOF (based on the Variant Effect Predictor) or missense variant of predicted damaging effect (based on SIFT/PolyPhen). Five of these 32 signals were further validated with functional experiments (Tables S6 and S7). Although the vast majority of signals (246 out of 278; 88.5%) were non-coding, only 17 such signals were followed up on experimentally (Figure 4B, Table S6).

*In silico* classification of protein-coding variants as either deleterious or benign is imperfect,^51^ and functional experiments at a given locus might not have assayed all statistically prioritized variants. Nonetheless, these approaches have the potential to improve understanding of the biological mechanisms and enable us to strategize interventions needed to mimic the effects of the protective alleles. For example, Sivanesan et al.^34^ experimentally validated that protective alleles of the IL23R protein display loss of function due to impairment of receptor maturation and reduced protein stability, suggesting antagonists of IL23R as a relevant therapeutic strategy for treating IBD. Therapeutic inhibition of IL23 is an approved treatment for psoriasis. Here, in agreement with the causal role of IL23 signaling in autoimmune diseases, GWAS and fine-mapping experiments suggest that strategies that directly inhibit the IL23 receptor (IL23R) could be of even greater potential therapeutic value.^34^ In another autoimmune GWAS locus, Richard et al.^35^ used allele-specific expression measurements to demonstrate that IBD-protective alleles in the chromosome 9q32 locus enhance expression of *TNFSF15* (MIM: 604052), suggesting agonists of TNFSF15 as potential therapies for treating autoimmune diseases. Similar examples of genes with fine-mapped loss- or partial-loss-of-function autoimmune-disease risk alleles included *IL18RAP* (MIM: 604509), *ITGAM* (MIM: 120980), *MST1* (MIM: 142408), *RNF186* (MIM: 617163), *TRAF3IP2* (MIM: 607043), and *CD58* (MIM: 153420),^32^^,^^35^, ^36^, ^37^^,^^41^^,^^45^^,^^46^^,^^48^ implying agonists of the protein products of these genes as potential therapeutic interventions (Figure 4C and Tables S6 and S7). *CARD9* (MIM: 607212), *NXPE1*, *PLCG2* (MIM: 600220), *SLAMF8* (MIM: 606620), *CCR6* (MIM: 601835), and *IRF5* (MIM: 607218) were among the genes that had loss- or partial-loss-of-function-protective alleles,^33^^,^^34^^,^^39^^,^^40^ predicting antagonists of the protein products of these genes as potential therapies (Figure 4D and Tables S6 and S7).

Genetic correlations across subsets of autoimmune diseases have been estimated from genetic-risk scores^52^ and LD score regression.^53^ Here, we asked a related question to estimate the extent of overlap in fine-mapping results across diseases at shared GWAS loci. We found that many loci implicated in more than one autoimmune disease displayed differences in the sets of prioritized variant(s) and/or direction of genotype effect on disease risk (e.g., Figure 4E, Table S6). Across loci with fine-mapping estimates for two or more autoimmune diseases, we found at least partial overlap among the prioritized variants in 41% of the pairwise comparisons (Figure 4F and Table S6). When the prioritized variant or variants were shared, we chose one variant to represent each independent signal and showed that the direction of effect was identical for 79.5% of the cases (Figure 4G and Tables S6 and S11). Although limited by the compiled fine-mapping manuscripts and not based on robust statistical signals such as evidence of a shared causal variant from pairwise colocalization analyses, these observations suggest that when the same locus is implicated in two or more autoimmune diseases, differences in the causal variants underlying each phenotype rather than differences in the direction of effects is the primary source of genetic discordance between different autoimmune phenotypes.

## Assigning confidence scores to estimates of candidate genes to identify most consistently dissected GWAS loci

**Figure 5:**
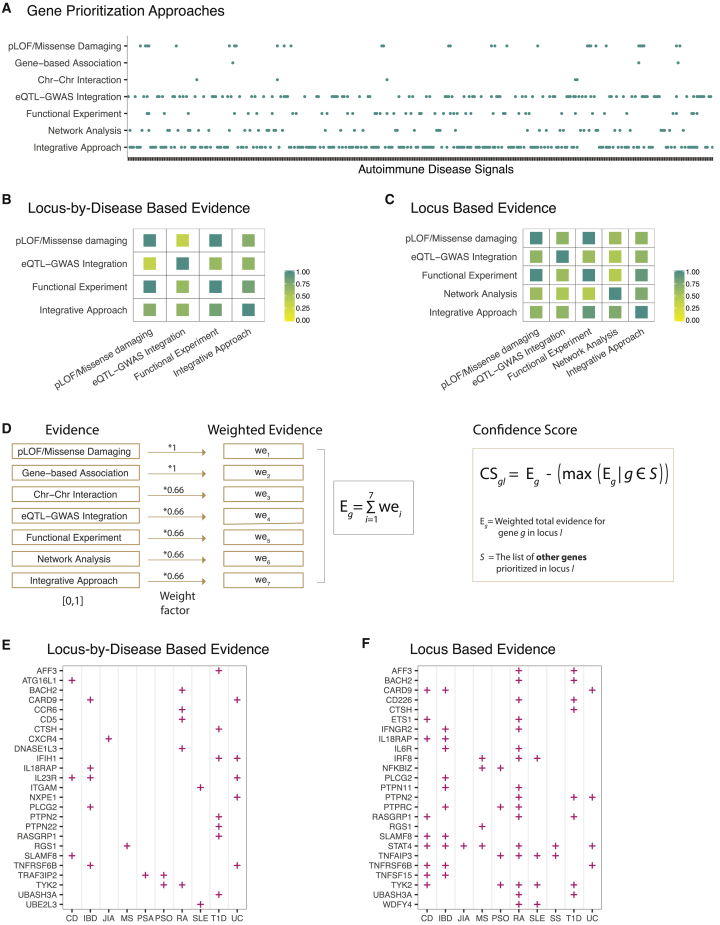
An integrated approach to assigning confidence scores to candidate-gene estimates (A) Gene-prioritization evidence compiled for 342 combinations of locus-by-disease association signals across 191 unique loci and 15 autoimmune diseases. Detailed information about each locus and fine-mapping evidence are included in Table S8. (B) This heatmap shows the frequency of at least one overlapping gene when the same locus-by-disease combination was dissected via multiple strategies. Results were plotted for pairwise strategies that co-dissected at least five locus-by-disease combinations. (C) This heatmap shows the frequency of at least one overlapping gene when the same autoimmune disease locus was dissected via multiple strategies. Results were plotted for pairwise strategies that co-dissected at least five autoimmune-disease loci. (D) Integration of gene-prioritization approaches to calculating confidence scores. (E) Top 25 prioritized genes when each autoimmune disease was considered individually (i.e., gene prioritization evidence of each disease was integrated separately even when multiple autoimmune diseases had GWAS signals and genes prioritized in the same locus). (F) Top 25 prioritized genes from disease agnostic analysis where gene-prioritization evidence across all autoimmune diseases was considered jointly in shared loci. Gene and disease names are sorted by alphabetical order. Abbreviations are as follows: CD, Crohn disease; IBD, inflammatory bowel disease; JIA, juvenile idiopathic arthritis; MS, multiple sclerosis; PSO, psoriasis; RA, rheumatoid arthritis; SLE, systemic lupus erythematosus; SS, systemic sclerosis; T1D, type 1 diabetes; and UC, ulcerative colitis.

GWAS have clearly shown that most complex-disease associations are driven by non-coding variants (Figure 4B). Although protein-coding variants can be readily linked to the genes that they affect, identifying the gene targets of non-coding variants has not been straightforward. Each GWAS locus often spans multiple genes, and non-coding variants do not necessarily regulate the closest gene. Additionally, gene regulation can be context and tissue specific, which adds an extra layer of complexity to identifying the gene involved and predicting the direction of gene regulatory effect on disease risk. After a GWAS, many genome-wide studies have assayed intermediate molecular traits including epigenetic marks, gene expression levels, and chromatin interaction datasets.^3^^,^^30^^,^^54^, ^55^, ^56^, ^57^, ^58^, ^59^, ^60^, ^61^, ^62^, ^63^, ^64^, ^65^, ^66^ An array of computational approaches have been developed to test the impact of genetic variation on these intermediate traits and to help researchers infer causal relationships between the molecular phenotypes and the downstream disease risk. Gene-based association tests of rare variants have been used to increase the statistical power and to enable direct identification of trait-relevant genes.^45^^,^^67^^,^^68^ Functions of the compelling candidate genes in GWAS loci have been tested with *in vitro* experiments and model organisms.^32^^,^^34^^,^^45^^,^^69^, ^70^, ^71^, ^72^, ^73^, ^74^, ^75^, ^76^, ^77^, ^78^, ^79^, ^80^, ^81^, ^82^, ^83^, ^84^, ^85^, ^86^, ^87^, ^88^, ^89^, ^90^, ^91^ Additionally, protein-protein interaction and co-expression networks have been integrated with GWAS results to both identify trait-relevant genes and pinpoint key network modules important in disease pathogenesis. In this study, we compiled candidate gene estimates from 342 combinations of locus-by-disease association signals across 191 unique loci and 15 autoimmune diseases (Table S8, Figure 5A). We had seven broad categories of gene-prioritization approaches (Figures 2 and 5A). We note that we only considered the results of studies that focused on identifying *cis* gene targets of GWAS variants. Approaches that focus on the *trans* gene targets or interacting pairs of the GWAS *cis* gene targets were beyond the scope of our study.

When the same association signal was dissected by different approaches, we found that there was often at least one overlap among the prioritized gene(s) (Figures 5B and 5C). Several integrative algorithms have been developed to rank genes in GWAS loci by incorporating results from different gene-prioritization approaches.^92^^,^^93^ These algorithms mainly rely on integrating results of genome-wide functional datasets such as genome-wide QTLs and chromatin interactions. However, they do not capture locus-specific evidence that often comes from GWAS follow-up studies that experimentally characterize one or a few GWAS loci.^32^, ^33^, ^34^, ^35^^,^^37^^,^^38^^,^^40^^,^^42^^,^^44^^,^^45^^,^^47^^,^^48^^,^^69^, ^70^, ^71^, ^72^, ^73^^,^^75^, ^76^, ^77^, ^78^, ^79^, ^80^, ^81^, ^82^, ^83^, ^84^, ^85^, ^86^, ^87^, ^88^^,^^90^^,^^91^ For example, while available integrative approaches would capture the colocalization signal between *BACH2* (MIM: 605394) eQTLs and type 1 diabetes, they would not integrate the results of a GWAS follow-up study that functionally linked the BACH2 protein to type 1 diabetes.^80^ Furthermore, a gene such as *CCR6* (MIM: 601835) could have been missed if locus-specific TALEN gene editing evidence in this locus had not been considered, as there is no reported statistical colocalization between the *CCR6* eQTLs and autoimmune-disease GWASs.^40^ Additionally, previously published integrative approaches^92^^,^^93^ allow prioritization of multiple genes in the same disease locus. Although it is biologically possible that multiple trait-relevant genes exist in the same locus, identifying loci where a single gene was consistently implicated by different approaches is crucial for drug-discovery scientists to prioritize among dozens or hundreds of loci with gene candidates. To address these key gaps for complex autoimmune diseases, we integrated the candidate gene predictions we compiled in a single framework. For each prioritized gene *g*, we calculated a weighted total evidence score (E_*g*_) as follows:

$${\text{E}}_{\text{g}}={\sum_{\text{i}=1}^{7}\text{we}}_{\text{i}},$$

where we_*i*_ corresponds to the weighted evidence that led to the prioritization of gene *g* on the basis of each gene-prioritization category *i*. We chose the weight factors of each gene-prioritization category *i* to reflect the consensus of confidence in evidence. The presence of predicted loss-of-function (pLOF) or missense damaging variant(s) among statistically prioritized variants in a GWAS locus is considered as the most direct evidence implying the trait-relevant genes. As such, we down-weighted all prioritization categories relative to “pLOF/missense damaging” and “gene-based association” categories (Figure 5D). For each prioritized gene *g* in each locus *l*, we then computed a confidence score (CS_*gl*_), which adjusts the total evidence score (E_*g*_) of each gene by the presence or absence of evidence supporting other genes (*S*) in the same locus:

$${\text{CS}}_{\text{gl}}={\text{E}}_{\text{g}}-\left(\max\left(E_{g}|\text{g∈s}\right)\right).$$

We applied this approach to each autoimmune disease separately (i.e., by calculating Eg scores based on evidence from individual diseases) (Table S9 and Figure 5E) and also performed a disease agnostic analysis where we considered gene prioritization evidence across all autoimmune diseases that had a GWAS signal in the same locus jointly (Table S10 and Figure 5F). Locus-based disease agnostic analysis can be advantageous in loci where a single gene underlies multiple autoimmune-disease associations. In contrast, this same approach can introduce noise to confidence scores when different genes in the same GWAS locus underlie different autoimmune diseases. Colocalization analyses^5^ across diseases could be used to guide use of disease agnostic approach. However, it is worth noting that the absence of a shared causal variant (either as a result of true absence or because of a lack of statistical power) does not rule out the presence of a shared trait-relevant gene underlying different autoimmune diseases. For example, different causal variants in distinct genomic elements might control regulation of the same gene to influence risk of multiple autoimmune diseases. As such, when a disease agnostic approach is used, a further comparison of the prioritized genes for each individual disease might be necessary to assess whether combining functional evidence could be justified or not. A comparison of the results of these two analyses (Tables S9 and S10) suggests that in most loci there is one candidate gene underlying different autoimmune diseases, and combining information across GWAS follow-up studies of different autoimmune phenotypes might help with the gene prioritization efforts. However, there are loci where different genes have been prioritized for different autoimmune diseases. For example, in the chromosome 6q27 locus, *CCR6* (MIM: 601835) was prioritized for rheumatoid arthritis,^40^^,^^55^ and *RNASET2* (MIM: 612944) was prioritized for Grave disease, vitiligo, and Crohn disease.^59^^,^^64^^,^^72^ Similarly, in the chromosome 11q12.2 locus, although *CD6* (MIM: 186720) was prioritized for multiple sclerosis and Crohn disease,^22^^,^^62^^,^^85^ *CD5* (MIM: 153340) was the gene prioritized for rheumatoid arthritis via multiple approaches.^20^^,^^55^

By applying both these strategies to the published GWAS follow-up evidence for 15 autoimmune diseases, we prioritized 189 candidate trait-relevant genes (Tables S9 and S10). Among the genes with top confidence scores were known drug target genes (e.g., *CD5* [MIM: 153340], *CXCR4* [MIM: 162643], *IL6R* [MIM: 147880], and *TYK2* [MIM: 176941]), genes with known immune-related functions (e.g., *IFIH1* [MIM: 606951], *IL18RAP* [MIM: 604509], *STAT4* [MIM: 600558], and *TNFAIP3* [MIM: 191163]), and genes that are lesser characterized in the context of autoimmune diseases (e.g., *AFF3* [MIM: 601464], *NXPE1*, and *UBE2L3* [MIM: 603721]). Although not definitive, the approach we used provided a straightforward means of integrating multiple sources of gene-prioritization evidence and allowed us to distinguish consistently dissected GWAS loci from those with conflicting results and/or multiple genes with similar degree of functional support.

## Discussion

Genetic variation underlies differences in disease pathogenesis. After the discovery of DNA, human geneticists largely focused on understanding how rare, large-effect size mutations can cause rare diseases that run in families with predictable inheritance patterns. Only with the development of DNA microarray and high-throughput sequencing technologies in the 2000s did it become possible to reveal the genetic architecture of common, complex diseases. Remarkable advances have been achieved in complex-trait genetics in the past 15 years. The current focus of many researchers in the field is to translate this growing genetic knowledge into therapeutics with the ultimate goal of reducing the burden of these diseases worldwide.

Human genetics has the potential to transform healthcare, and it is imperative that this transformation impact all affected human lives equally. Yet, there is an unfortunate lack of diversity in the subjects included in GWASs,^94^ most of which are based on populations of European ancestry. There are many reports indicating that lack of ethnic diversity in human genetic studies might lead to inaccurate predictions of disease risk and lack of interventions in under-studied populations.^94^ Moreover, trans-ethnic analyses of GWAS results across diverse populations can take advantage of population-specific LD patterns and narrow down disease-causing variants that are shared between populations.^7^^,^^8^ Despite studies reporting that associations of SNPs with autoimmune diseases are largely consistent across populations,^95^^,^^96^ we were unable to find autoimmune trans-ethnic studies that fully and equally captured human genetic variation across different populations. Specifically, in most trans-ethnic studies, genotyping arrays used in non-European populations were designed for capturing genetic variation in Europeans. Hence, these studies were reporting a rather incomplete and potentially biased picture. As research moves forward, we see great need and promise in carefully designed GWAS and fine-mapping studies in diverse human populations. Such studies not only will uncover the full genetic architecture underlying human disease but also have the potential to improve the risk prediction and precision in medical care.

Another major obstacle that we encountered while reviewing GWAS fine-mapping studies was the difficulty of summarizing the results from the HLA locus. Genetic variation in the HLA locus represents the strongest predisposing signal for almost all autoimmune diseases^97^ and yet it is practically impossible to review the fine-mapping evidence in this locus in a systematic way. Among the characteristic features of the HLA locus are the high degree of polymorphism and strong LD. As such, fine mapping in this locus is by default challenging. An additional layer of complexity is added when fine-mapping studies in this locus report their association results at different molecular resolutions (i.e., haplotype level, amino acid level, and nucleotide level). Direct conversions between different molecular resolutions are not always possible. There are also many multi-allelic variants with different numbers of distinct alleles in the HLA locus. A commonly ignored potential issue in HLA fine-mapping studies is that association p values could be inflated by the degree of complexity of the variants.^21^ Therefore, p values attributed to different models with variable variant complexities could be misleading during prioritization of causal variants. Clinical laboratories have a relatively long history of using classical HLA alleles at different resolutions depending on the patient needs. As the field moves forward, we anticipate that at least within the GWAS fine-mapping community, there will be better-standardized ways to capture, report, and analyze the variation in this complex and important locus.

Matching therapeutic modality to genetic mechanism of action has the potential to facilitate the translational research in a way that had not been possible before GWASs.^98^ For protein-coding variants with predicted damaging effects, it is relatively straightforward to determine whether the risk or the protective allele has the deleterious effect on the protein function. The genetic mechanism of action for non-coding variants is often predicted with gene expression levels as an intermediate phenotype between genotype and the disease outcome. Although such approaches prioritize trait-relevant genes more accurately than baselines, interpretation of these analyses could be convoluted.^99^ First, true or artifactual co-regulation might lead to multiple gene prioritizations within the same locus. Second, even when a single gene is prioritized in a locus, the allelic direction of effect on gene expression might be tissue specific. Hence, it might not be possible to infer a therapeutic strategy if the mechanistically disease-related tissue is unknown or when genotype effect on gene expression is opposite in two potentially disease-relevant tissues. We expect that overcoming these challenges will involve expansion of current gene-expression panels to cover additional cell and stimulation types as well as methodologies that focus on identifying the relevant cell type for each disease or each locus-by-disease combination. Additionally, revealing causal intermediate phenotypes (eg., blood cell indices) of complex diseases could improve the inference on causal variants and genes in GWAS loci, in particular when fine-mapping signals of disease and their underlying intermediate phenotypes are considered jointly. Until all loci are fine-mapped and genetic-based therapeutic modalities are reliably predicted, we see great benefit in allocating resources to the most consistently dissected loci where a single gene is clearly and consistently prioritized on the basis of multiple sources of functional evidence.

In the very early stages of GWASs, Hindorff et al. realized the importance of forming a catalog of GWAS results, manually curated the published SNP-trait associations, and built the commonly used NHGRI GWAS Catalog.^100^ As a collective effort and largely in a project-centric model, researchers then started sharing full summary statistics to follow up on and fine-map primary GWAS findings. Ongoing efforts include standardizing heterogeneous formats and harmonizing content of GWAS summary statistics.^1^ The availability of a harmonized, downloadable catalog of GWAS summary statistics has facilitated many aspects of GWAS fine-mapping studies. Here, in parallel to these efforts, we present a systematic approach cataloging and summarizing published GWAS fine-mapping evidence for autoimmune diseases. We believe the work presented here could expedite the process of translating GWAS findings into therapeutics and be expanded into collaborative efforts to catalog and evaluate published fine-mapping evidence for all complex human diseases.

## Declaration of interests

M.C. is an employee of Bristol-Myers Squibb. J.C.M. is an employee and a shareholder of Bristol-Myers Squibb. C.D.B. declares no competing interests.

## Data and code availability

The code generated during this study is available at https://github.com/minalc44/autoimmune/. Supplemental information includes all datasets analyzed during this study.
